# Purinergic smooth muscle contractions in the human prostate: estimation of relevance and characterization of different agonists

**DOI:** 10.1007/s00210-020-02044-4

**Published:** 2021-01-11

**Authors:** Annabel Spek, Bingsheng Li, Beata Rutz, Anna Ciotkowska, Ru Huang, Yuhan Liu, Ruixiao Wang, Frank Strittmatter, Raphaela Waidelich, Christian G. Stief, Martin Hennenberg

**Affiliations:** 1 Department of Urology, University Hospital, LMU Munich, Munich, Germany; 2 Urologische Klinik und Poliklinik, Marchioninistr. 15, 81377 Munich, Germany

**Keywords:** Lower urinary tract symptoms (LUTS), Benign prostatic hyperplasia (BPH), Smooth muscle contraction, Purinergic receptors, P2X, P2Y

## Abstract

Non-adrenergic prostate smooth muscle contractions may account for the limited effectiveness of α_1_-adrenoceptor antagonists, which are the first-line option for medical treatment of voiding symptoms suggestive of benign prostatic hyperplasia. In non-human prostates, purinergic agonists induce contractions reaching similar magnitudes as α_1_-adrenergic contractions. However, evidence for the human prostate is highly limited, and pointed to much weaker purinergic contractions. Here, we examined contractions of different purinergic agonists in human prostate tissues. Tissues were obtained from radical prostatectomy. Contractions were studied in an organ bath, and expression of purinergic receptors was studied by RT-PCR. Electric field stimulation (EFS)–induced contractions amounted to 104% of KCl-induced contractions (95% CI: 84–124%). From all tested agonists, only ATP induced concentration-dependent contractions, reaching an average maximum of 18% (12–24%) of KCl. Maximum tensions following application of other agonists averaged to 7.1% of KCl for α,β-methylene-ATP (1.8–12.4%), 3.9% for β,γ-methylene-ATP (2.0–5.4%), 3.1% for 2-methylthio-ATP (− 0.1–6.3%), and 5.1% for ATPγS (1.0–9.2%). Responses were not affected by the P2X antagonist NF023 or the P2Y antagonist PPADS. mRNA expression of P2X1-4 correlated with expression of a marker for catecholaminergic nerves, although neither ATP, NF023, nor PPADS changed EFS-induced contractions. Correlation between expression of receptors and the smooth muscle marker calponin was not observed. Our findings point to a low relevance of purinergic contractions in the human prostate, compared to other contractile stimuli in the human prostate and compared to purinergic contractions in non-human prostates. Purinergic contractions in the human prostate are not sensitive to NF023 or PPADS.

**Supplementary Information:**

The online version contains supplementary material available at 10.1007/s00210-020-02044-4.

## Introduction

Prostate smooth muscle contraction can be induced by activation of α_1_-adrenoceptors, and in parallel by non-adrenergic mediators (Hennenberg et al. 2014b). Elevated prostate smooth muscle tone may contribute to urethral obstruction in benign prostatic hyperplasia (BPH), resulting in impairments of urinary flow and bladder emptying, and finally in lower urinary tract symptoms (LUTS) suggestive of BPH (Hennenberg et al. 2014b). Accordingly, prostate smooth muscle contraction is an important target for medical treatment of voiding symptoms in BPH, which includes α_1_-adrenoceptor antagonists (α_1_-blockers) as the first-line option and the phosphodiesterase-5 inhibitor tadalafil as an alternative (Hennenberg et al. 2014b; Oelke et al. 2013). Drugs of both classes inhibit prostate smooth muscle contraction (Michel and Vrydag 2006; Uckert and Kuczyk 2011). In particular, α_1_-blockers are supposed to improve voiding symptoms in BPH by widening of the urethra and reduction of intraurethral pressure, due to prostate smooth muscle relaxation (Caine et al. 1976; Oelke et al. 2013). However, the effectiveness of these drugs is clearly limited. α_1_-Blockers improve symptom scores and the maximum urinary flow rate (*Q*_max_) by maximally 50% and 40%, while even placebos improve symptom scores and *Q*_max_ up to 30% and 15%, respectively (Chapple et al. 2011; Dahm et al. 2016; Eredics et al. 2017; Kortmann et al. 2003; Madersbacher et al. 2007; Strand et al. 2017; Wang et al. 2014). Improvements of symptom scores by tadalafil are in comparable ranges to α_1_-blockers (Pattanaik et al. 2018). In contrast to α_1_-blockers, however, it did not improve *Q*_max_ in most trials (Pattanaik et al. 2018).

In parallel to activation of α_1_-adrenoceptors by adrenergic neurotransmission, prostate smooth muscle contractions can be induced by non-adrenergic mediators. In the human prostate, the so far best-characterized non-adrenergic mediators of smooth muscle contraction are endothelins, which induce contractions of similar magnitudes as α_1_-adrenoceptors, and thromboxane A_2_, inducing contractions with forces amounting roughly to one-third of adrenergic contractions (Hennenberg et al. 2017). Non-adrenergic prostate smooth muscle contractions are resistant to α_1_-blockers, what may account at least for the limited efficacy of α_1_-blockers (Hennenberg et al. 2017; Hennenberg et al. 2013). Thus, it has been proposed that non-adrenergic contractions may preserve prostate smooth muscle tone, urethral obstruction, and symptoms despite treatment with α_1_-blockers (Hennenberg et al. 2017; Hennenberg et al. 2014b). Consequently, understanding of non-adrenergic prostate smooth muscle contractions and adequate knowledge regarding the composition of prostate smooth muscle tone in human BPH are of high clinical relevance and are essential for the development of future options for treatment of male LUTS.

Adenosine-5′-triphosphate (ATP) induces smooth muscle contractions in the lower urinary tract, cardiovascular system, and gastrointestinal tract, by activation of purinergic receptors (Burnstock and Kennedy 2011; Ford and Cockayne 2011; Ralevic 2015). Purinergic contractions involve activation of purinergic P2X receptors, mainly P2X1 located on smooth muscle cells. Postsynaptic P2X1 receptors on smooth cells are activated by ATP released by sympathic neurotransmission, resulting in contraction by depolarization and calcium influx (Burnstock and Kennedy 2011; Ralevic 2015). In addition, purinergic contractions may include prejunctional mechanisms, where activation of P2X receptors located on sympathic neurons modulates neurotransmitter release and subsequent neurogenic smooth muscle contractions (Burnstock and Kennedy 2011; Ralevic 2015). Besides P2X receptors, ATP activates P2Y receptors, which may be involved in purine- and pyrimidine-mediated control of smooth muscle tone as well, including P2Y-induced vasocontractions or bladder smooth muscle contraction and relaxation (Kira et al. 2017; Pinna et al. 2005; Tengah et al. 2018; Yu et al. 2013). Purinergic prostate smooth muscle contraction has been repeatedly claimed and shown for animal prostates. However, data for human prostate smooth muscle are highly limited to date and contrast the few available data from non-human prostates (Hennenberg et al. 2017; Wang et al. 2020). Thus, to clarify the relevance of purinergic human prostate smooth muscle contractions, we here examined effects of ATP and of different available P2X and P2Y agonists and antagonists on smooth muscle contraction of human prostate tissues.

## Materials and methods

### Human prostate tissues

Human prostate tissues were obtained from patients who underwent radical prostatectomy for prostate cancer (*n* = 132). Patients with previous transurethral resection of the prostate were excluded. This study was carried out in accordance with the Declaration of Helsinki of the World Medical Association and has been approved by the ethics committee of the Ludwig-Maximilian University, Munich, Germany. Informed consent was obtained from all patients. All samples and data were collected and analyzed anonymously. Following removal of prostates from patients, macroscopic pathologic examination and sampling were performed within approximately 30 min. Organ bath studies were started within 1 h following sampling, i.e., approximately 1.5 h following surgical removal of the organs. For molecular analyses, tissues were directly shock frozen in liquid nitrogen following arrival in the laboratory, i.e., again approximately 1.5 h following surgical removal of the organs. For transport and storage, organs and tissues were stored in Custodiol^®^ solution (Köhler, Bensheim, Germany). For macroscopic examination and sampling of prostate tissues, the prostate was opened by a single longitudinal cut reaching from the capsule to the urethra. Subsequently, both intersections were checked macroscopically for any obvious tumor infiltration. Tissues were taken solely from the periurethral zone, considering the fact that most prostate cancers arise in the peripheral zone (Pradidarcheep et al. 2011; Shaikhibrahim et al. 2012). In fact, tumor infiltration in the periurethral zone (where sampling was performed) was very rare (found in less than 1% of prostates). Prostates showing tumors in the periurethral zone upon macroscopic inspection were not subjected to sampling and were not included in this study. BPH is present in ca. 80% of patients with prostate cancer (Alcaraz et al. 2009; Orsted and Bojesen 2013).

### Porcine interlobar arteries

Kidneys from pigs were obtained from a local slaughterhouse, where animals were sacrificed during the night. Subsequently, organs were picked up by a local butcher, stored at 4 °C, and transferred from the butcher’s shop (Metzgerei Brehm, Planegg, Germany) to the laboratory in the early morning. Here, segments of interlobar arteries were immediately prepared from organs, and cut into rings (3–4 mm in length) after removing adipose and connective tissue around the vessels. Subsequently, vessel segments were transferred to Custodiol^®^ solution, and stored at 4 °C in the Custodiol solution until use for organ bath experiments. Experiments were started not later than 3 h following the preparation of vessels.

### Organ bath

Prostate strips (6 × 3 × 3 mm) were mounted in 10 ml aerated (95% O_2_ and 5% CO_2_) tissue baths (Danish Myotechnology, Aahus, Denmark) with four chambers, containing Krebs-Henseleit solution (37 °C, pH 7.4). Preparations were stretched to 4.9 mN and left to equilibrate for 45 min. In the initial phase of the equilibration period, spontaneous decreases in tone are usually observed. Therefore, tension was adjusted three times during the equilibration period, until a stable resting tone of 4.9 mN was attained. After the equilibration period, the maximum contraction induced by 80 mM KCl was assessed. Subsequently, chambers were washed three times with Krebs-Henseleit solution for a total of 30 min, and antagonists or an equivalent amount of water for controls was added. Cumulative concentration response curves for ATP or agonists or frequency response curves for electric field stimulation (EFS) were constructed 30 min after addition of antagonists or water. Application of EFS simulates action potentials, resulting in the release of endogenous neurotransmitters, including noradrenaline. To examine effects of ATP on EFS-induced contractions, a single dose of ATP or an equivalent amount of water for controls was added, and EFS was applied as soon as an obviously maximum ATP-induced contraction was obtained, or after a corresponding period in controls. Effects of antagonists or ATP on EFS-induced contractions were examined in experiments using samples from the same prostate in each experiment. Thus, from each prostate, samples were allocated to the control, antagonist, or ATP groups within the same experiment, so that the same prostates were used in both groups of each series. Wherever possible, double determinations were performed. From a total of 101 organ bath experiments performed with agonists or EFS using prostate tissues, this was possible in 59 experiments for control groups, and in 52 experiments for antagonist, inhibitor, or ATP groups. The application of water and antagonist or ATP to chambers was changed for each experiment. Only one curve was recorded with each sample. For the calculation of agonist-induced contractions, tensions were expressed as percentage of 80 mM KCl-induced contractions, as this may correct different stromal/epithelial ratios and different smooth muscle content, resulting from varying phenotypes and degrees of BPH, or from any other individual variation and heterogeneity between prostate samples and patients.

As ATP was applied as disodium salt and in concentrations up to 30 mM, several control experiments were performed. Thus, pH values were determined in samples (25 ml) of Krebs-Henseleit solution after adding the maximum concentration of ATP disodium salt (30 mM), or after adding a corresponding amount of water. In a next series, samples (25 ml) of Krebs-Henseleit solution were titrated using 2 M hydrochloric acid (HCl), until the same pH was obtained as observed in samples containing 30 mM ATP. Next, an amount of HCl required to obtain this pH value was applied to human prostate tissues in organ bath experiments, 30 min following return to a stable baseline after a first KCl-induced contraction and washout of KCl, and being followed by a second KCl-induced contraction after washout (three times, total 20 min) of HCl. In separate, further control experiments, effects of additional sodium chloride (NaCl, plus 60 mM to NaCl in standard Krebs-Henseleit solution) or of sodium sulfate (Na_2_SO_4_, final concentration 30 mM) were examined, corresponding to the sodium content of the ATP salt. These experiments followed the same design as described for HCl, only that HCl was replaced by the sodium salts. Each of these experiments was repeated five times, using tissues from five different prostates; however, in doing so, a total of five prostates was used for all three series of control experiments, i.e., tissues from five prostates were allocated to three series with HCl, NaCl, and Na_2_SO_4_. Each of these experiments was based on double determinations (i.e., two channels). Tensions after application of HCl or sodium salt are expressed as percentage of tension raised by the second application of KCl.

Procedures in experiments with porcine interlobar arteries were similar to procedures in experiments with human prostate tissues. As P2X and P2Y agonists failed to induce obvious contractions in human prostate tissues (with the exception of ATP), these experiments were performed to assess whether these agonists induce contractions of other smooth muscle preparations under our conditions. For pretension, segments of interlobar arteries were stretched to 9.8 mN. Tension was adjusted three times during the equilibration period, until a stable resting tone of 9.8 mN was attained. After the equilibration period, maximum contraction induced by 80 mM KCl was assessed. As soon as a plateau contraction induced by KCl was obtained, chambers were washed three times with Krebs-Henseleit solution for a total of 30 min, and cumulative concentration response curves for agonists were constructed. In each experiment, all four chambers of one organ bath were filled with segments from the same vessel, and only one concentration response or frequency response curve was recorded with each segment. From these four channels, two were examined with one agonist and two others with another agonist. For each agonist, five independent experiments were performed, i.e., tissues from five animals were examined by double determinations. Agonist-induced tensions were expressed as percentage of 80 mM KCl-induced contractions. In fact, normalization to KCl allows to examine possible alterations of receptor responsiveness, while correlations between agonist-induced force and ring weight, length, or cross-sectional area are weak or lacking in organ bath experiments using vessel segments (Erdogan et al. 2020).

### RT-PCR

RNA from frozen prostate tissues was isolated using the RNeasy Mini kit (Qiagen, Hilden, Germany). For isolation, 30 mg of tissues was homogenized using the FastPrep^®^-24 system with matrix A (MP Biomedicals, Illkirch, France). RNA concentrations were measured spectrophotometrically. Reverse transcription to cDNA was performed with 1 μg of isolated RNA using the Reverse Transcription System (Promega, Madison, WI, USA). RT-PCR for P2X receptors, calponin, cytokeratin 19, tyrosine hydroxylase (TH), prostate-specific antigen (PSA), and glyceraldehyde 3-phosphate dehydrogenase (GAPDH) was performed with a Roche Light Cycler (Roche, Basel, Switzerland) using primers provided by Qiagen (Hilden, Germany) as ready-to-use mixes, based on the RefSeq accession numbers NM_002558 for P2RX1, NM_012226 for P2RX2, NM_002559 for P2RX3, NM_001256796 for P2RX4, NM_ 002562 for P2RX7, NM_001299 for calponin-1, NM_000360 for TH, NM_002276 for cytokeratin-19, and NM_001030047 for KLK3 (synonymous PSA), and NM_001256799 for GAPDH. PCR reactions were performed in a volume of 25 μl containing 5 μl LightCycler^®^ FastStart DNA MasterPlus SYBR Green I (Roche, Basel, Switzerland), 1 μl template, 1 μl primer, and 18 μl water. Denaturation was performed for 10 min at 95 °C, and amplification with 45 cycles of 15 s at 95 °C followed by 60 s at 60 °C. The specificity of primers and amplification was demonstrated by subsequent analysis of melting points, which revealed single peaks for each target. Results were expressed using the ΔCt method, where number of cycles (Ct) at which the fluorescence signal exceeded a defined threshold for GAPDH was subtracted from Ct values for targets (Ct_target_-Ct_GAPDH_ = ΔCt), and values were calculated as 2^−ΔCt^. Finally, 2^−ΔCt^ values were normalized to the mean of all values obtained using different tissues for the same targets, resulting in reported “relative 2^−ΔCt^” values.

### Western blot analyses

Frozen tissues were homogenized in a buffer containing 25 mM Tris/HCl, 10 μM phenylmethanesulfonyl fluoride, 1 mM benzamidine, and 10 μg/ml leupeptine hemisulfate, using the FastPrep^®^-24 system with matrix A (MP Biomedicals, Illkirch, France). After centrifugation (20,000 *g*, 4 min), supernatants were assayed for protein concentration using the Dc-Assay kit (Biorad, Munich, Germany) and boiled for 10 min with sodium dodecyl sulfate (SDS) sample buffer (Roth, Karlsruhe, Germany). Samples (20 μg/lane) were subjected to SDS-polyacrylamide gel electrophoresis, and proteins were blotted on Protran^®^ nitrocellulose membranes (Schleicher & Schuell, Dassel, Germany). Membranes were blocked with phosphate-buffered saline (PBS) containing 5% milk powder (Roth, Karlsruhe, Germany) overnight and incubated with mouse monoclonal anti pan-cytokeratin (sc-8018), mouse monoclonal anti calponin 1/2/3 (sc-136987), mouse monoclonal anti PSA (sc-7316), and mouse monoclonal anti-β-actin antibody (sc-47778) (all from Santa Cruz Biotechnology, Dallas, TX, USA). Primary antibodies and secondary peroxidase-coupled antibody were diluted in PBS containing 0.1% Tween 20 (PBS-T) and 5% milk powder. Membranes were washed with PBS-T after any incubation with primary or secondary antibodies or biotin-HRP. Finally, blots were developed with enhanced chemiluminescence (ECL) using ECL Hyperfilm (GE Healthcare, Freiburg, Germany).

### Data and statistical analyses

Data in concentration response curves are presented as means ± standard deviation (SD) with the indicated number (*n*) of independent experiments. Maximum contractions in groups without pharmacological intervention were collected from each experiment using human prostate tissues, and compiled for each agonist across different series for an overall comparison of maximum, agonist-induced contractile forces. These values observed across different series are presented as means with 95% confidence interval (CI), calculated using the SPSS^®^ version 20 (IBM SPSS Statistics, IBM Corporation, Armonk, NY, USA) and shown in scatter plots. The present study and analyses were designed to be exploratory but not designed to test a pre-specified statistical null hypothesis. Despite the exploratory design of this study, the (minimum) number of experiments and group sizes in organ bath experiments was pre-planned as *n* = 5/group. Thus, all groups were based on five or more independent experiments and included tissues from five or more patients in each group. Data were extracted and analyzed, after at least five experiments of a series were performed. Following this analysis, series with prostate tissues were discontinued if no effect was expected on this basis, or if an inhibition was observed after these experiments. If the initial results were unclear, e.g., due to lacking contractions in some experiments, series were continued. For experiments with interlobar arteries, group sizes were preset to *n* = 5 independent experiments. In line with the exploratory character of our study, with the design of our procedures, and with recent recommendations for data analysis (Motulsky 2014), but also as apparent inhibitions of contractions were either not observed or became very obvious (tetrodotoxin, tamsulosin) without statistical tests, application of statistical tests and reporting of *p* values were omitted. Exceptions are Spearman’s correlation analyses (without p values), which were performed using GraphPad Prism 6 (Statcon, Witzenhausen, Germany). In experiments based on antibody-based detection, case numbers were adapted to technical settings (lanes in gel electrophoresis). According to the paired design (allocation of samples from each tissue to the control and antagonist groups), groups being compared with each other showed identical group sizes. No data or experiments were excluded from analyses. To some extent, diagrams intending to visualize results from correlation analysis of receptor and marker expression are an exception. These included only relative 2^−ΔCt^ values ≤ 4, as correlations would not become obvious from diagrams, if all values > 4 were included in this format. However, indicated *r* values are based on all available values.

### Materials, drugs, and nomenclature

If not other stated, drugs described below were obtained from Tocris (Bristol, UK). Adenosine-5′-triphosphate (ATP) is the endogenous ligand of P2X receptors (Alexander et al. 2019b), and was obtained from Sigma (Munich, Germany). Aqueous suspensions of ATP disodium salt (500 mM) were freshly prepared before each experiment and stored on ice until application to organ bath chambers. α,β-Methylene-adenosine 5′-triphosphate trisodium salt (α,β-methylene-ATP) is a full P2X1 and P2X3 agonist, but may also cause rapid desensitization and consequent inhibition of P2X1- and P2X3-mediated effects (Alexander et al. 2019b). β,γ-Methyleneadenosine 5′-triphosphate disodium salt (β,γ-methylene-ATP) is a full P2X1 agonist (Alexander et al. 2019b), and was obtained from Sigma (Munich, Germany). 2-Methylthio-adenosine-5′-triphosphate tetrasodium salt (2-methylthio-ATP) is an agonist of P2Y1, -6, -11 and -13, and also antagonist of P2Y1, with EC_50_ and *K*_i_ values ranging between 20 and 200 μM (Alexander et al. 2019a). The non-hydrolyzable ATP analog adenosine 5′-(3-thio)triphosphate tetralithium salt (ATPγS) is an agonist of P2Y1, -11 and -13, with EC_50_ and *K*_i_ values ranging between 40 and 13 μM (Alexander et al. 2019a). 8,8′-[Carbonylbis(imino-3,1-phenylenecarbonylimino)]bis-1,3,5-naphthalene-trisulphonic acid, hexasodium salt (NF023) is a P2X antagonist, with an IC_50_ of 200 nM for human P2X1, but 29 to > 100 μM for human P2X2-, P2X3-, and P2X4-mediated responses (Alexander et al. 2019b; Soto et al. 1999). Pyridoxalphosphate-6-azophenyl-2′,4′-disulfonic acid tetrasodium salt (PPADS) is an antagonist of P2Y1, -4, -6 and -13, with *K*_i_ and *K*_B_ values ranging between 6 and 100 μM for P2Y1, -6 and -13, and EC_50_ values ranging between 10 and 10 mM for P2Y4 (Alexander et al. 2019a). Stock solutions (10 mM) of α,β-methylene-ATP, β,γ-methylene-ATP, 2-methylthio-ATP, ATPγS, NF023, and PPADS were prepared with water, and stored at − 20 °C until used and before further dilution with water. Tetrodotoxin (TTX) is a sodium channel inhibitor, which inhibits neurotransmitter release and subsequent neurogenic contractions in smooth muscle preparations, including human prostate tissues (Angulo et al. 2012; Li et al. 2020b). Stock solutions (1 mM) were prepared with water, and stored at − 20 °C until used. Tamsulosin (5-[(2R)-2-[2-(2-ethoxyphenoxy)ethylamino]propyl]-2-methoxybenzenesulfonamide) is an α_1_-adrenoceptor antagonist, with high selectivity for the α_1A_ subtype (Alexander et al. 2019a; Oelke et al. 2013). Stock solutions (10 mM) were prepared in DMSO, and further diluted (1:100, to 100 μM) with water and stored at − 20 °C until use.

## Results

### Maximum contractions

**Fig. 1:**
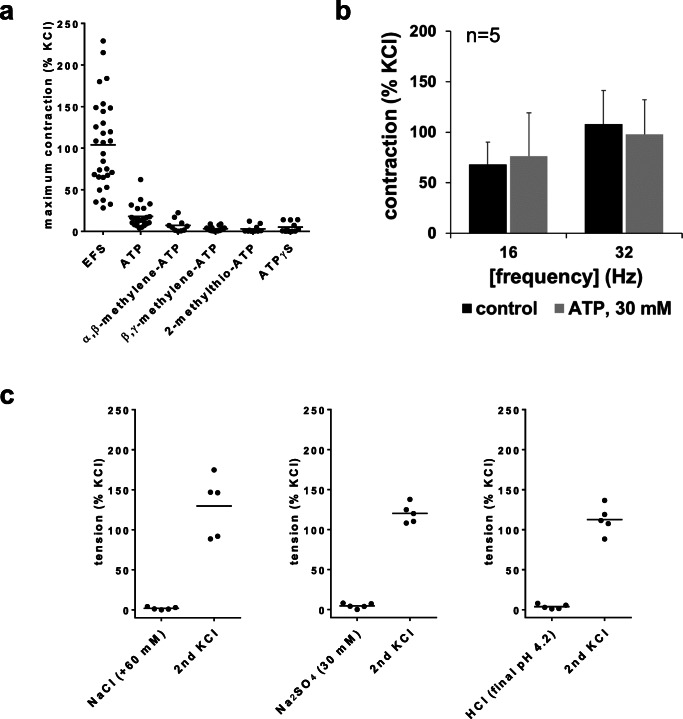
Maximum EFS- and agonist-induced contractions (**a**), effects of ATP disodium salt on EFS-induced contractions (**b**), and control experiments (**c**) in human prostate tissues. In **a**, maximum contractions and tensions from each single experiment (i.e., each prostate) in control groups of all series of organ bath experiments (Figs. 1b, and 2, 3, 4, and 5) are plotted, regardless at which frequency or agonist concentration this occured. In **b**, EFS-induced contractions were induced after a stable, i.e., maximum contraction was attained following administration of 30 mM ATP disodium salt, or after a corresponding period following administration of an equivalent amount of water. In **c**, NaCl, Na_2_SO_4_, or HCl was applied in amounts required for elevations of NaCl concentrations of 60 mM, required to obtain the final Na_2_SO_4_ concentrations of 30 mM, or required to reduce the pH to 4.2 (corresponding to the pH measured after application of 30 mM ATP disodium salt). Salt- and HCl-induced tensions were referred to a second KCl-induced contraction (raised as control for intact tissue at the end of the experiment), which is again plotted and expressed as percentage of the first KCl-induced contraction (raised in the beginning of the experiment). Shown are all values from single experiments in **a**, means ± SD from series with prostate tissues from *n* = 5 patients in **b**, and all values from single experiments from series with prostate tissues from *n* = 5 patients in **c**, with tissue from one prostate being allocated to the control and ATP groups in one of a total of five independent experiment in **b**

EFS, ATP, and different ATP analogs were applied to prostate tissues. Across different series and without any further pharmacological intervention (i.e., without adding antagonists or inhibitors), EFS-induced contractions amounted to 104% of KCl-induced contractions (95% CI: 84–124%) at the highest applied frequency of 32 Hz (Fig. 1a). ATP induced contractions with an average maximum of 18% of KCl-induced contractions (95% CI: 12–24%) (Fig. 1a). Within the applied concentration range (1–30 mM), maximum ATP-induced contractions occurred mostly at 30 mM (in 18 of a total of 23 prostates challenged with ATP), and at 10 mM in some cases (5 of 23 prostates challenged with ATP). All other examined agonists virtually failed to induce obvious contractions, or a concentration dependency of contractions was lacking. Specifically, maximum tensions following application of agonists (each 0.1–30 μM) averaged to 7.1% of KCl-induced contractions for α,β-methylene-ATP (95% CI: 1.8–12.4%), 3.2% for β,γ-methylene-ATP (95% CI: 1.6–4.9%), 3.1% for 2-methylthio-ATP (95% CI: − 0.1–6.3%), and 5.0% for ATPγS (95% CI: 1.0–9.2%) (Fig. 1a).

Even the strongest contractions resulting from purinergic agonists, observed in single tissues, remained weaker than average EFS-induced contractions, or even stayed below the lowest EFS-induced contractions (Fig. 1a). Strongest responses ranged between 28 and 62% of KCl-induced contractions for ATP (in 6 of 16 prostates, where contractions were induced by ATP), 17 and 22% of KCl for α,β-methylene-ATP (2 of 10), 7.5 and 9% of KCl for β,γ-methylene-ATP (4 of 16), 5 and 12% of KCl for 2-methylthio-ATP (3 of 10), and 7 and 14% of KCl for ATPγS (4 of 11) (Fig. 1a).

As ATP was applied as a disodium salt and in concentrations up to 30 mM, several series of control experiments were performed. To estimate, whether ATP-induced contractions may result from elevations of sodium concentrations, two different sodium salts were applied to prostate tissues in amounts corresponding to the sodium content of 30 mM ATP disodium salt. In separate series, neither increases of sodium chloride (plus 60 mM, additional to sodium chloride contained in Krebs-Henseleit solution) nor application of sodium sulfate (30 mM) resulted in contractions of prostate tissues (Fig. 1c). To estimate whether pH changes may account for contractions observed after 30 mM ATP disodium salt, pH changes in the Krebs-Henseleit solution and tensions after corresponding pH adaptions in organ bath chambers were examined. A 30-mM ATP decreased the pH of the Krebs-Henseleit solution to 4.2 (95% CI: 4.18–4.21), compared to a pH of 7.5 (95% CI: 7.48–7.5) after addition of a corresponding amount of water. In organ bath experiments, application of hydrochloric acid in an amount required to reduce the pH to 4.2 did not result in contractions of prostate tissues (Fig. 1c).

### Effects of ATP on EFS-induced contractions

To examine the effects of ATP on EFS-induced contractions, EFS (16 and 32 Hz) was applied as soon as a maximum tone was obtained following application of a single bolus of ATP (final concentration 30 mM), or following a corresponding period after an equivalent amount of water (controls). At both frequencies, tensions after EFS were similar following EFS in controls (= EFS + water) and following ATP before EFS (ATP + EFS) (Fig. 1b), indicating that EFS- and ATP-induced contractions are not additive.

### Effects of NF023 and PPADS on ATP-induced contractions

**Fig. 2:**
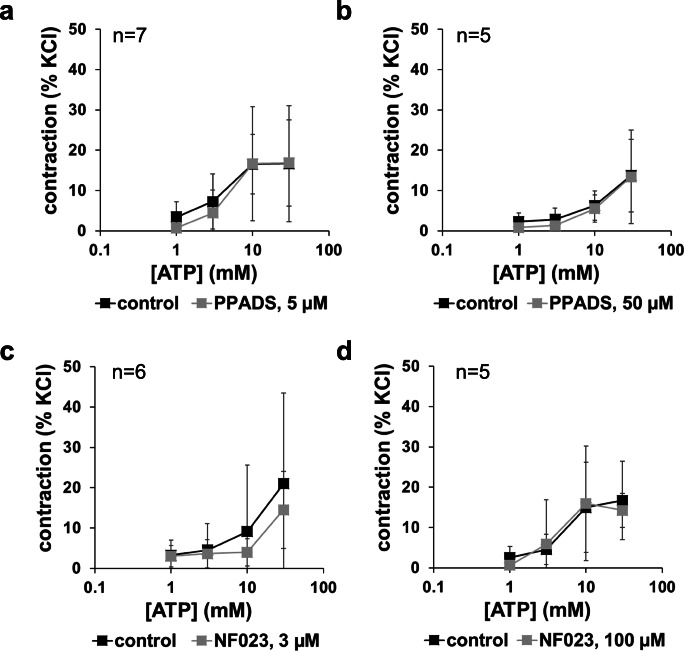
Concentration-dependent contractions by ATP disodium salt, and effects of NF023 (3 μM, 100 μM) and PPADS (5 μM, 50 μM) in human prostate tissues. Construction of concentration response curves was started 30 min following administration of antagonists, or of an equivalent amount of water (solvent) in controls. Shown are means ± SD from four independent series, each with prostate tissues from *n* = 7 (**a**), *n* = 5 (**b**), *n* = 6 (**c**), and *n* = 5 (**d**) different patients, where tissue from each prostate was allocated to the control and antagonist groups in each single experiment

Applied in a range of 1 to 30 mM, ATP induced concentration-dependent contractions. In two from four independent series performed with prostates from different patients, concentration response curves showed a sigmoidal character, which occured in both the control and the antagonist groups (Fig. 2). The P2Y antagonist PPADS (5 μM, 50 μM) or the P2X antagonist NF023 (3 μM, 100 μM) did not change concentration response curves for ATP (1–30 mM) (Fig. 2). Any effect on maximum contractions, or on EC_50_ values for ATP, did not become apparent (Fig. 2).

### Effects of NF023 and PPADS on EFS-induced contractions

**Fig. 3:**
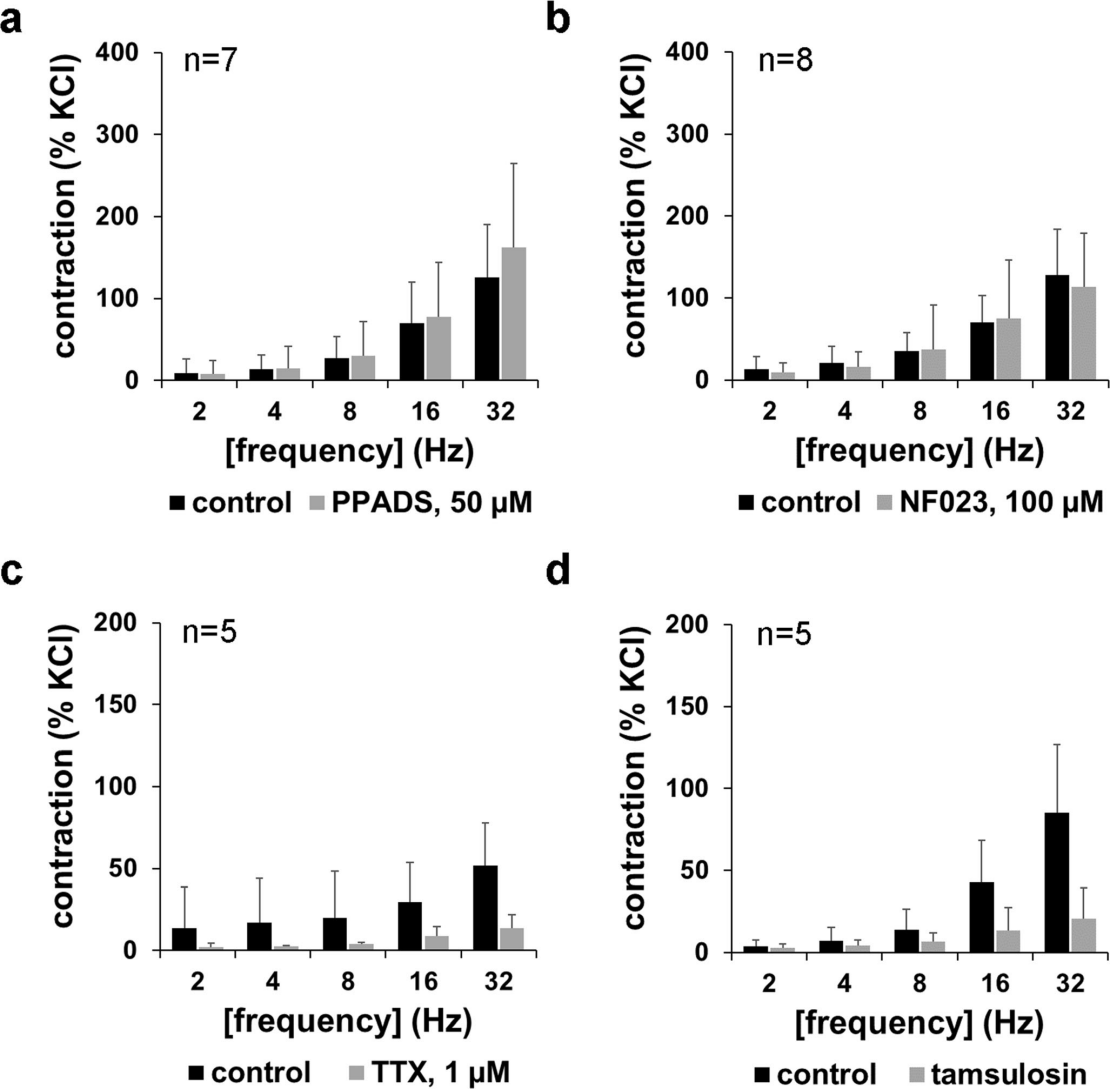
Frequency-dependent contractions by EFS, and effects of NF023 (100 μM), PPADS (50 μM), tetrodotoxin (TTX) (1 μM), and tamsulosin (300 nM) in human prostate tissues. Construction of frequency response curves was started 30 min following the administration of antagonists or TTX, or of equivalent amounts of solvents in controls. Shown are means ± SD from four independent series, each with prostate tissues from *n* = 7 (**a**), *n* = 8 (**b**), *n* = 5 (**c**), or *n* = 5 (**d**) different patients, where tissue from each prostate was allocated to the control and antagonist groups in each single experiment

PPADS (50 μM) or NF023 (100 μM) did not cause any apparent changes in EFS-induced contractions (Fig. 3a, b). Any effect on maximum contractions, or at any frequency or on the EC_50_ of frequency-dependent contractions, did not become apparent (Fig. 3a, b).

### Effects of TTX and tamsulosin on EFS-induced contractions

To confirm that EFS-induced contractions were neurogenic and susceptible to pharmacologic interventions under our conditions, effects of the sodium channel inhibitor TTX (1 μM) and of the α_1_-adrenoceptor antagonist tamsulosin (300 nM) were examined in separate series. Both drugs caused profound inhibitions of EFS-induced contractions (Fig. 3c, d).

### α,β-Methylene-ATP- and β,γ-methylene-ATP-induced contractions

**Fig. 4:**
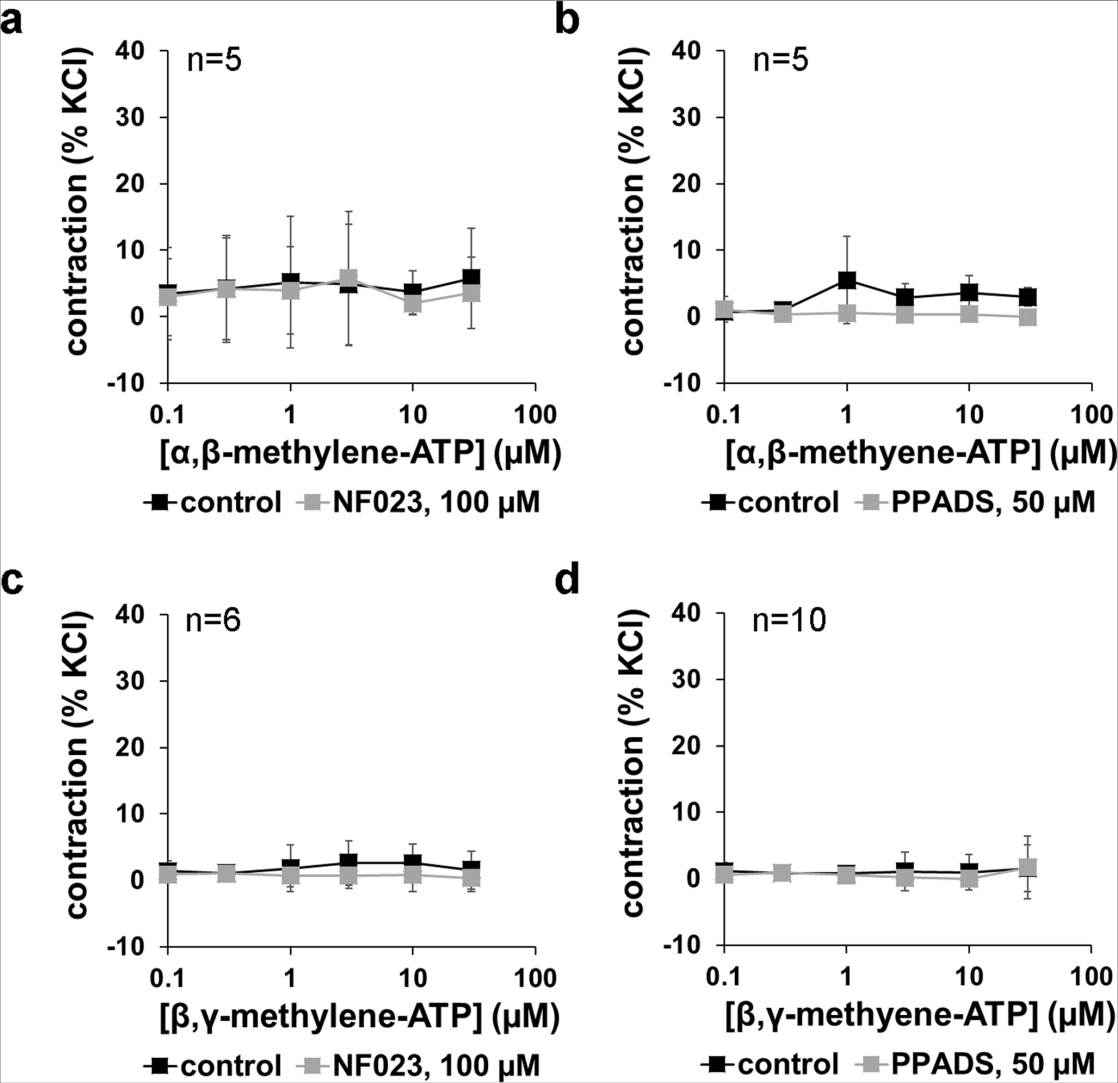
Effects of α,β-methylene-ATP and β,γ-methylene-ATP, with and without preceding administration of NF023 (100 μM) and PPADS (50 μM) on tension of human prostate tissues. Application of α,β-methylene-ATP or β,γ-methylene-ATP in cumulative concentrations was started 30 min following administration of NF023 or PPADS, or of equivalent amounts of water (solvent) in controls. Shown are means ± SD from four independent series, each with prostate tissues from *n* = 5 (**a**), *n* = 5 (**b**), *n* = 6 (**c**), and *n* = 10 (**d**) different patients, where tissue from each prostate was allocated to the control and antagonist groups in each single experiment

Applied in a range of 0.1 to 30 μM, α,β-methylene-ATP and β,γ-methylene-ATP did not induce concentration-dependent contractions (Fig. 4). For both agonists, two independent series were performed using PPADS and NF023, using prostate tissues from different patients, each containing paired control and antagonist groups. Maximum average tensions did not exceed 6% of KCl-induced contractions at any of the agonist concentrations in any group of any of the four series (Fig. 4). PPADS (50 μM) or NF023 (100 μM) did not cause any apparent changes in tensions (Fig. 4).

### 2-Methylthio-ATP- and ATPγS-induced contractions

**Fig. 5:**
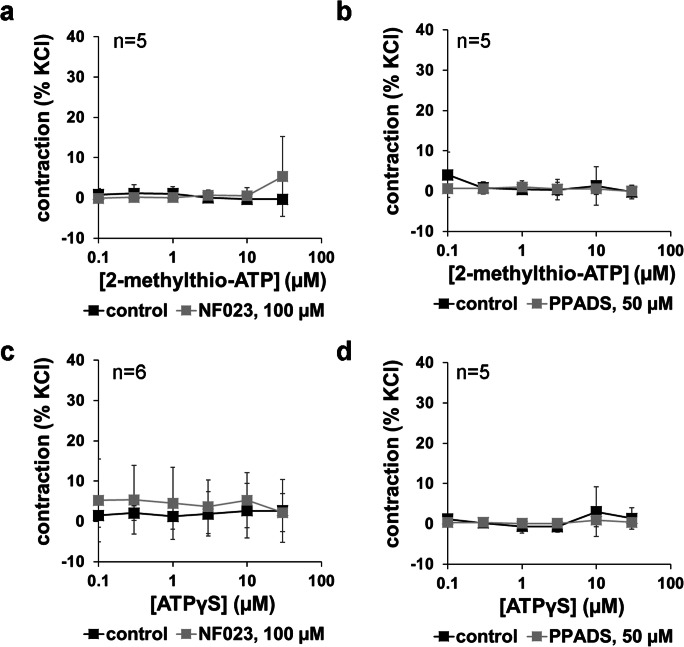
Effects of ATPγS and 2-methylthio-ATP, with and without preceding administration of NF023 (100 μM) and PPADS (50 μM) on tension of human prostate tissues. Application of ATPγS or 2-methylthio-ATP in cumulative concentrations was started 30 min following administration of NF023 or PPADS, or of equivalent amounts of water (solvent) in controls. Shown are means ± SD from four independent series, each with prostate tissues from *n* = 5 (**a**), *n* = 5 (**b**), *n* = 6 (**c**), and *n* = 5 (**d**) different patients, where tissue from each prostate was allocated to the control and antagonist groups in each single experiment

2-Methylthio-ATP and ATPγS were applied in a range of 0.1 to 30 μM and did not induce concentration-dependent contractions (Fig. 5). For both agonists, two independent series were performed using PPADS and NF023, using prostate tissues from different patients, each containing paired control and antagonist groups. Maximum average tensions did not exceed 8% of KCl-induced contractions at any of the agonist concentrations, in any group of any of the four series (Fig. 5). PPADS (50 μM) or NF023 (100 μM) did not cause any apparent changes in tensions (Fig. 5).

### Agonist-induced contractions of renal interlobar arteries

**Fig. 6:**
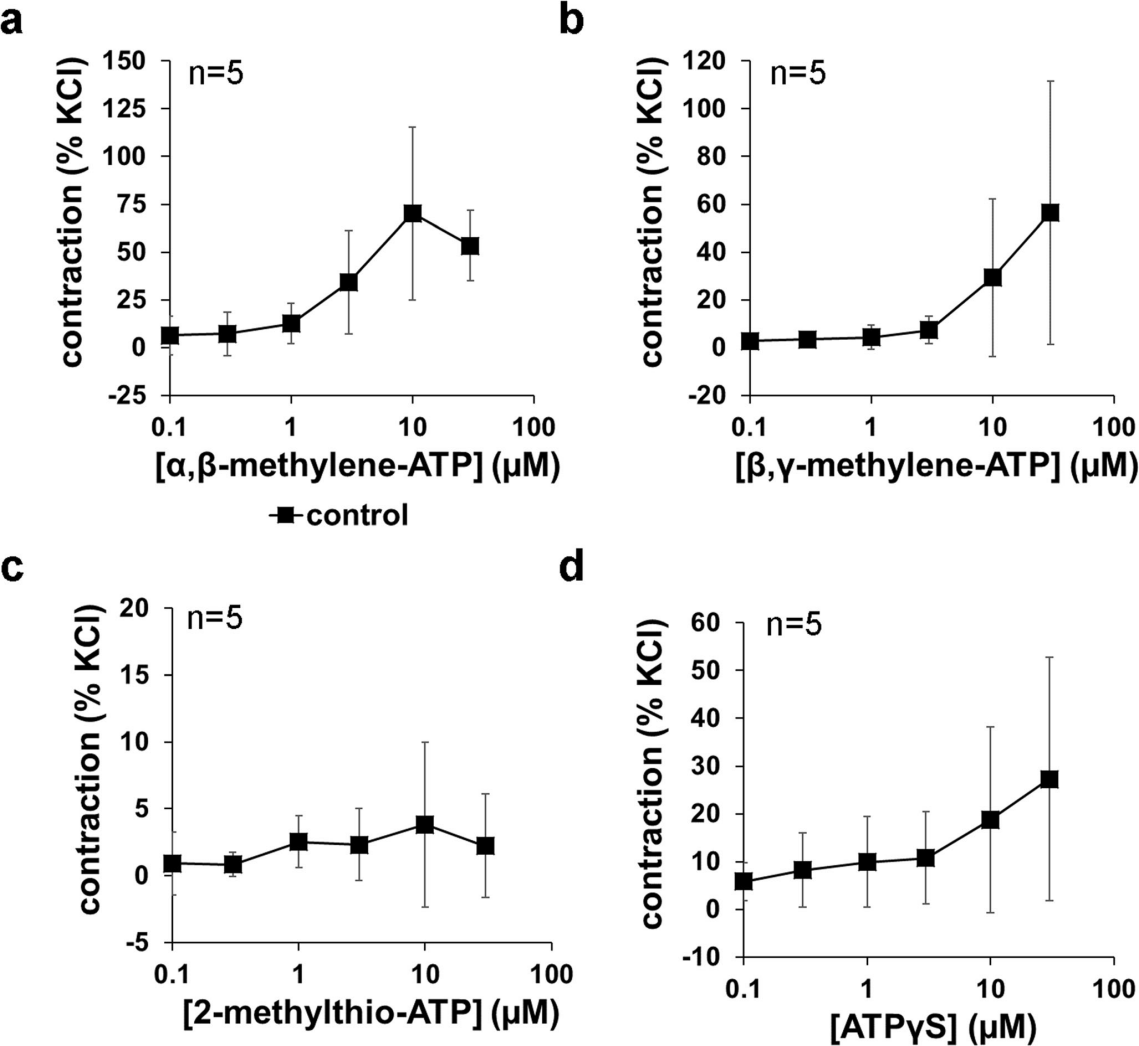
Effects of α,β-methylene-ATP, β,γ-methylene-ATP, 2-methylthio-ATP, and ATPγS on tension of renal interlobar arteries from pig kidneys. Application of agonists in cumulative concentrations was started 30 min following washout of high molar KCl and return to a stable baseline. Shown are means ± SD from four independent series, each with tissues from *n* = 5 different animals

α,β-Methylene-ATP, β,γ-methylene-ATP, 2-methylthio-ATP, and ATPγS were applied to interlobar arteries from porcine kidneys using the same concentration ranges as applied to human prostate tissues (Fig. 6). In contrast to prostate tissues, α,β-methylene-ATP, β,γ-methylene-ATP, and ATPγS induced obvious contractions of interlobar arteries (Fig. 6). Averages of maximum contractions amounted to 73% of KCl-induced contractions (95% CI: 20 to 126%) for α,β-methylene-ATP (maximum contractions occurring at 10–30 μM), 68% of KCl (1.7 to 135%) for β,γ-methylene-ATP (occurring at 10–30 μM), and 30% of KCl (1.8 to 57%) for ATPγS (occurring at 10–30 μM). 2-Methylthio-ATP did not induce obvious contractions of interlobar arteries (Fig. 6). Averages of maximum tensions following application of 2-methylthio-ATP amounted to 4.9% of KCl-induced contractions (− 2.0 to 11.8%) (occurring at 0.1–10 μM).

### P2X detection by RT-PCR and marker expression

**Fig. 7:**
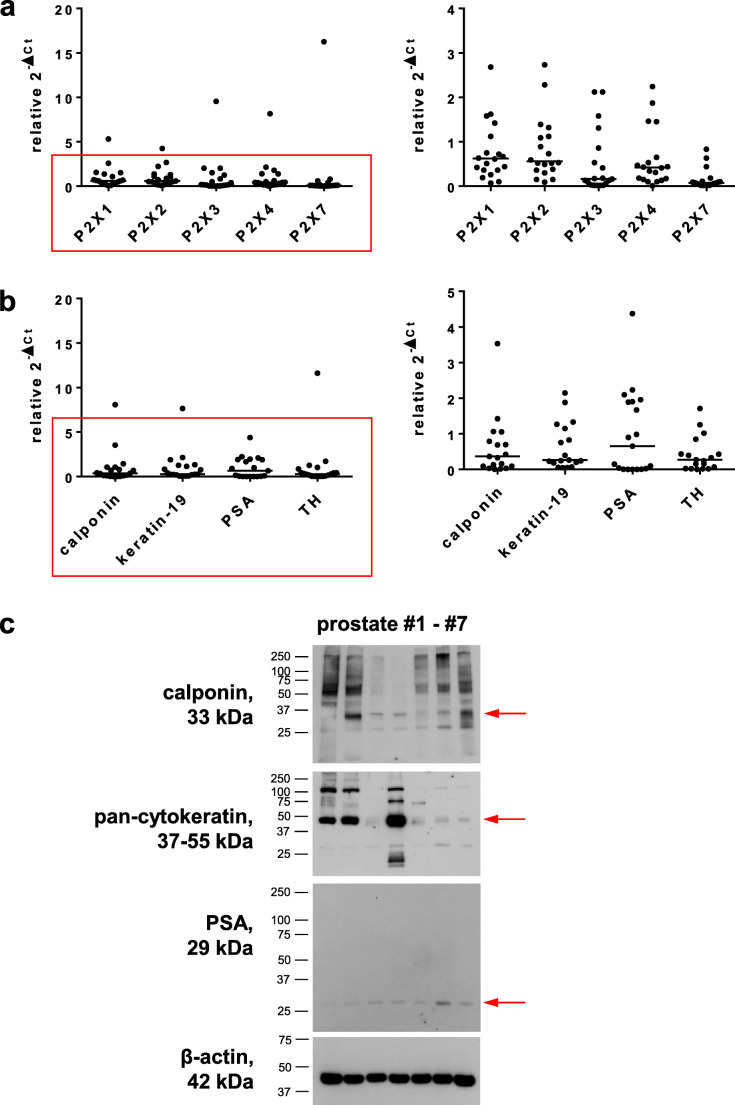
Detection of P2X1, P2X2, P2X3, P2X4, and P2X7 by RT-PCR, and of markers by RT-PCR and western blot in human prostate tissues. In **a** and **b**, single values (relative 2^−ΔCt^, i.e., 2^−ΔCt^ of each sample normalized to the mean of all values for the corresponding target) for all examined P2X subtypes (**a**), and all examined markers (**b**). In the left panels, all single values for all tissues examined by RT-PCR (from 19 patients) are shown, while single values not exceeding a relative 2^−ΔCt^ of 4 (**a**) or 5 (**b**) are shown in right panels (= cutout from left panels, to visualize variability). In **c**, western blot analyses were performed for markers and β-actin with prostate tissues from seven patients. Shown are complete blots (except for β-actin) from all included samples. Arrows indicate the expected molecular weights of proteins (calponin, PSA), or the presumed position of keratin-19 (pan-cytokeratin). Positions of marker bands are indicated at the left side of each blot (sizes in kDa). Samples and arrangement of their order was the same for each blot. Calponin was detected as a marker for smooth muscle cells, keratin-19 and pan-cytokeratin as markers for glandular epithelial cells, tyrosin hydroxylase as a marker for catecholaminergic nerves, and PSA as a marker for degree of BPH

mRNA levels of five P2X subtypes and four markers were semiquantitatively compared by RT-PCR between prostate tissues from 19 patients. All examined P2X receptor subtypes (P2X1, P2X2, P2X3, P2X4, P2X7) were detectable in each sample (Fig. 7a). Detection of calponin as a marker for smooth muscle cells, keratin-19 as a marker for glandular epithelial cells, tyrosine hydroxylase as a marker for catecholaminergic nerves, and PSA as a marker for degree of BPH suggested variations in expression levels of markers, reflecting variations in tissue composition and divergent degree of BPH between samples from different patients (Fig. 7b). The heterogenous character of tissues suggested by RT-PCR was confirmed by western blot analysis of prostate tissues from seven patients, where contents of calponin, cytokeratins, and PSA obviously varied (Fig. 7c).

**Fig. 8:**
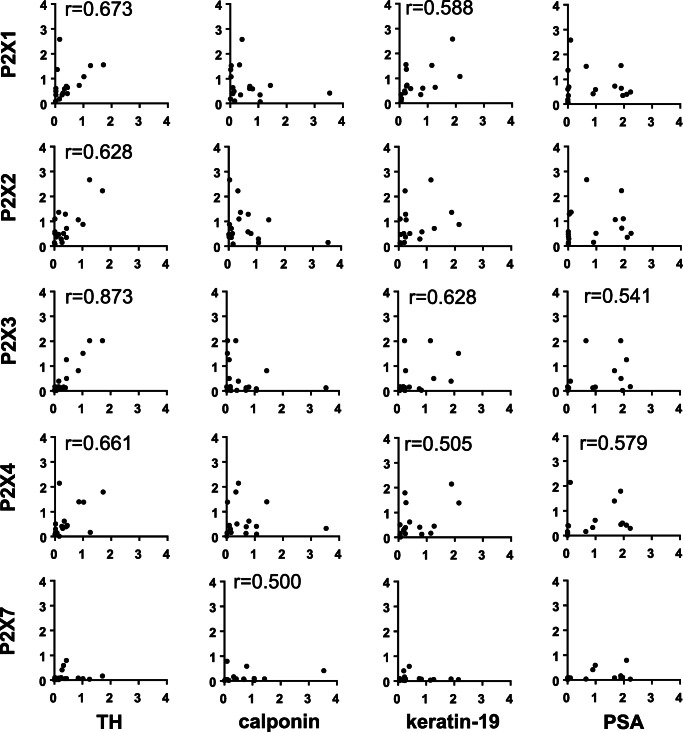
Spearman’s correlation analyses were performed by between mRNA expression of all examined P2X subtypes, and markers (tyrosine hydroxylase (TH) for catecholaminergic nerves, calponin-1 for smooth muscle, cytokeratin-19 for glandular epithelial cells, PSA for degree of BPH). Diagrams only include values not exceeding 4 (all values are relative 2^−ΔCt^), to realize visual presentation of correlations in addition to *r* values. All values for P2X receptors, including those > 4, are shown in Fig. 7a. Indicated *r* values refer to complete data sets, including values > 4

Detection of receptors and markers by RT-PCR in the same samples suggested positive correlations (*r* > 0.5) of expression levels of P2X1, P2X2, P2X3, and P2X4 with expression levels of tyrosine hydroxylase (Fig. 8). No certain correlations were observed between expression of receptors and calponin (Fig. 8). Expression of P2X1, P2X3, and P2X4 correlated positively with keratin-19 (Fig. 8). Expression of P2X3 and P2X4 correlated positively with PSA (Fig. 8), reflecting their upregulation with degree of BPH.

## Discussion

Previous studies reported purinergic smooth muscle contractions of non-human prostates, with contractile forces commonly approaching ranges of neurogenic and α_1_-adrenergic contractions. Consequently, a high relevance of ATP-induced contractions for composition of prostate smooth muscle tone has been assumed (Hennenberg et al. 2014a), even before the first data became available for human prostate smooth muscle (Wang et al. 2020). To the best of our knowledge, previous data describing purinergic contractions of human prostate smooth muscle are limited to one data set, which suggested much lower contractions than reported for non-human prostates (Wang et al. 2020). These conflicting findings from human and non-human prostates, together with the limited evidence available from human prostates, are contrasted by the assumed high relevance, and prompted us to address purinergic smooth muscle contractions of human prostate tissues more in detail. Our current findings suggest a rather low relevance of ATP-induced contractions in the human prostate, which were insensitive to P2X and P2Y antagonists, while several other P2X and P2Y agonists did not induce relevant contractions at all.

Maximum ATP-induced contractions in control groups were roughly similar across four independent series, where tissues from different patients were used for each series. Maximum contractions averaged to 18% of high molar KCl-induced contractions. This is similar to recently reported, and to the best of our knowledge, single available values for human tissues, where ATP-induced contractions amounted to an average maximum of 14% of KCl-induced contractions, under identical conditions (Wang et al. 2020). Previously reported values for ATP-induced contractions of tissues from rodent prostates are considerably higher than these values from human prostate tissues, even though only indirect comparisons are possible. In mouse prostate tissues, maximum ATP-induced contractions amounted to approximately 50% and 70% of high molar KCl-induced contractions (declining with age, i.e., 12 month and 8 weeks) (White et al. 2015). In other studies, ATP-induced contractions were not referred to KCl, but indirect comparisons are possible by comparison to EFS- or noradrenaline-induced contractions across different series, and determined within the same studies. Thus, in rat prostates, maximum ATP-induced contractions amounted to approximately 85% and 66% of noradrenaline-induced contractions (Brandli et al. 2010; Xu and Ventura 2010). In another study using rat prostate tissues, comparison was possible to EFS-induced contractions across different series, suggesting that maximum ATP-induced contractions amounted to approximately 65% of EFS-induced contractions (Ventura et al. 2003). Reported data for purinergic contractions of guinea pig prostates are hardly comparable, and include average maximum ATP-induced contractions of 0.1 g, while a single, representative experiment pointed to an EFS-induced contraction of 1.1 g (Buljubasich and Ventura 2004).

EFS-induced contractions in our current study are in the same range as in our previous studies using human prostate tissues, where EFS- and noradrenaline-induced contractions mostly ranged around and between 80 and 150% of KCl-induced contractions (Hennenberg et al. 2018; Hennenberg et al. 2013; Wang et al. 2020; Yu et al. 2018). Similar relationships may be strongly assumed for prostate tissues from rodent models. Considering that α_1_-blockers represent the first-line option for medical treatment of voiding symptoms in BPH (Oelke et al. 2013), α_1_-adrenergic and EFS-induced contractions may be considered a gold standard of prostate smooth muscle contraction. Together, this may impart a certain conclusiveness of the indirect comparisons made here between our current and previous studies, between human and non-human tissues, and between purinergic and non-purinergic contractions.

Using the purinergic agonists α,β-methylene-ATP and β,γ-methylene-ATP, we observed only weak or no contractions at all. Apart from the 2 examined prostates, where α,β-methylene-ATP induced contractions of 16 and 22% of KCl-induced contractions, tensions after application of these agonists never exceeded 10% in our study, what may be hardly distinguished from the resting tone. For both agonists, previous data are to the best of our knowledge not available for human prostate tissues. Findings for both agonists in our study are contrasted by divergent findings from non-human prostates. In mouse prostate tissues, maximum α,β-methylene-ATP-induced contractions amounted to approximately 70% and 85% of high molar KCl-induced contractions (at an age of 12 months and 8 weeks) (White et al. 2015). In rat prostate tissues, maximum α,β- and β,γ-methylene-ATP-induced contractions amounted to approximately 80% and 100% of EFS-induced contractions (Ventura et al. 2003). Another study reported values suggesting contractions of rat prostate tissues induced by 10 μM α,β-methylene-ATP amounting to approximately 53% of phenylephrine-induced or 64% of EFS-induced contractions (Calmasini et al. 2015). Reported data for guinea pig prostates are again hardly conclusive, as they include average maximum contractions of 0.14 g using α,β-methylene-ATP and 0.25 g using β,γ-methylene-ATP, but only a single, representative experiment again suggesting an EFS-induced contraction of 1.1 g (Buljubasich and Ventura 2004). Data for other animal species are to the best of our knowledge available neither for ATP nor for other purinergic agonists.

ATPγS and 2-methylthio-ATP may act via P2Y receptors and have been rarely applied to prostate tissues. In guinea pig prostates, 2-methylthio-ATP induced contractions of 0.1 g, compared to a single, representative experiment pointing to an EFS-induced contraction of 1.1 g (Buljubasich and Ventura 2004). Therefore, and considering that PPADS did not inhibit ATP- or EFS-induced contractions, we assume that P2Y receptors play no or only a minor role in the control of smooth muscle tone in the human prostate. In line with these findings, previous data suggested no contractions of human prostate tissues by the P2Y agonist uridine-adenosine-5′-tetraphosphate (UP(4)A) (Hennenberg et al. 2017).

To exclude that lacking contractions of prostate tissues after application of α,β- and β,γ-methylene-ATP, ATPγS and 2-methylthio-ATP were attributed to technical reasons, we applied these agonists to arterial tissues. α,β-Methylene-ATP, β,γ-methylene-ATP, and ATPγS induced an obvious contraction of renal interlobar arteries from pig kidneys, confirming that these agonists generally worked under our conditions. Purinergic vascular smooth muscle contractions have been previously reported for different vessel types and species (Ralevic 2015), although to the best of our knowledge not for isolated, interlobar arteries. However, and in line with our findings obtained from isolated interlobar arteries, purinergic pressure responses of the intrarenal vasculature have been suggested by in situ–perfused rodent kidneys (Rump et al. 1990; Vonend et al. 2005).

Several studies performed with non-human prostates suggested that ATP may be released as a cotransmitter in adrenergic neurotransmission and may contribute to subsequent α_1_-adrenergic contraction of prostate smooth muscle (Buljubasich and Ventura 2004; Lam et al. 2016; Ventura et al. 2003). In line with the contrasting results for different species, divergent findings have been reported also regarding the overall function of ATP in prostate smooth muscle contraction. In addition to ATP-induced contractions, inhibition of EFS-induced contractions by ATP has been reported as well for rat prostate tissues (Preston et al. 2000). An amplification or inhibition of EFS-induced contractions by ATP in the human prostate appears unlikely, as we did not observe an effect of ATP on EFS-induced contractions in our current study.

EFS-induced and purinergic contractions in our study were insensitive to the P2X antagonist NF023 and the P2Y antagonist PPADS. Based on previously reported IC_50_, *K*_i_, and *K*_B_ values and the concentrations applied in our experiments, an effect on purinergic contractions should be expected, if these receptors were involved. As none of both antagonists affected ATP-induced contractions, it may be supposed that ATP-induced smooth muscle contractions in the human prostate take place receptor-independently. As ATP was applied as a disodium salt and using concentrations up to 30 mM, we performed control experiments excluding that elevations of sodium concentrations or changes in pH accounted for the ATP-induced contractions. On the other hand, our control experiments did not allow conclusions whether changes in pH after application of disodium ATP may impair contraction levels, i.e., accounted for the low levels of ATP-induced contractions. In addition to smooth muscle contractions in non-human prostates, inhibition of contractions by α,β-methylene-ATP has been reported, which has been explained by P2X receptor desensitization resulting P2X receptor activation by α,β-methylene-ATP (Lam et al. 2016). Rapid desensitization may theoretically account for lacking contractile effects in our study, but did obviously not occur in interlobar arteries under our conditions.

To confirm that contractions under our conditions were sensitive to pharmacologic interventions, we demonstrate inhibition of EFS-induced contractions by TTX and tamsulosin. Consequently, we exclude that lacking effects of NF023 and PPADS in our experiments represent artifacts or are attributed to technical reasons. Thus, if contractions in our samples were susceptible to the applied antagonists, this should be seen under our conditions. TTX is known to inhibit EFS-induced prostate smooth muscle contractions by inhibition of endogenous neurotransmission (Angulo et al. 2012; Li et al. 2020b). In turn, tamsulosin is an α_1_-adrenoceptor antagonist, which is commonly applied for treatment of LUTS suggestive of BPH, and inhibits EFS-induced contractions by preventing the activation of α_1_-adrenoceptors located on prostate smooth muscle cells by neurogenically released noradrenaline (Hennenberg et al. 2014b; Oelke et al. 2013). The magnitude of EFS-induced contractions (without TTX or tamsulosin) is in similar ranges as reported in our previous studies, performed under the same conditions (Hennenberg et al. 2018; Hennenberg et al. 2013; Wang et al. 2020; Yu et al. 2018). Therefore, we exclude that the low contractile forces induced by ATP and the lacking contractions after application of other agonists result from an overall reduced contractility of samples in the present study. Finally, we exclude that low or lacking contractions in our study were attributed to too low concentrations, as we applied similar ranges or even higher concentrations as in previous studies.

Our study was performed using human prostate tissues from patients undergoing radical prostatectomy for prostate cancer. It has been estimated that 80% of patients with prostate cancer may show BPH (Alcaraz et al. 2009; Orsted and Bojesen 2013). Certain diagnosis of BPH requires histological analyses, as BPH occurs not infrequently without enlargement of the prostate (Abrams 1994; Lepor 2004). Due to limited sample sizes and anonymization immediately after sampling, BPH was not histologically assessed in our samples and reference to routine pathological diagnoses (or any other patients’ characteristics, e.g., age) was not possible in our study. However, taking the high prevalence of BPH in patients with prostate cancer into account and considering that samples were taken from the periurethral zone, where BPH is most prominent (Pradidarcheep et al. 2011), we assume that most tissues included here showed BPH. On the other hand, BPH appears highly heterogenous, as BPH may be either epithelial or stromal, i.e., occurs as increased glandular compartments in some patients, but by stromal growth in other patients (Strand et al. 2017). Further sources of heterogeneity between tissues are divergent degree of BPH and any other individual variation. Consequently, tissue composition and smooth muscle content may vary, what may alter contractility. In our study, heterogeneity of tissue composition is reflected by varying contents of calponin, cytokeratin, and tyrosine hydroxylase, while different PSA contents reflect different degrees of BPH. Similar variations have been shown in our previous studies, where tissues were collected under the same conditions (Hennenberg et al. 2018; Li et al. 2020a; Yu et al. 2018). To correct different overall contractilities resulting from divergent smooth muscle content of tissues, we normalized agonist- and EFS-induced contractions to high molar KCl-induced contractions, as recommended recently (Erdogan et al. 2020).

Detection by RT-PCR was performed for P2X subtypes 1, 2, 3, 4, and 7, as smooth muscle contraction in different organs and different studies has been previously attributed to these subtypes. In particular, P2X1 has been suspected to be expressed on smooth muscle cells of different smooth muscle–rich organs and to account for purinergic contractions. As we did not observe certain correlations between mRNA levels of P2X1 and calponin, our findings may hardly support the concept that P2X1 is generally expressed in human prostate smooth muscle cells. Correlation analyses demonstrated that P2X1–4 mRNAs increase with the content of tyrosine hydroxylase, what may reflect expression in catecholaminergic nerves. In fact, P2X receptors located on neurons may be involved in the regulation of smooth muscle contraction by prejunctional mechanisms, including purinergic regulation of neurotransmitter release (Ralevic 2015). However, in our study, this is contrasted by lacking effects of ATP, NF023, and PPADS on neurogenic, EFS-induced contractions. Together with findings from organ bath experiments, our results from mRNA detection suggest non-contractile functions of purinergic receptors, which may include functions in glandular epithelial cells, or upregulation of P2X3 and P2X4 in BPH. On the other hand and considering that tissues from some few patients responded with moderate contractions to α,β- and β,y-methylene-ATP, it could be speculated that upregulation of P2X receptors (e.g., to the highest mRNA levels seen in RT-PCR) imparts responsiveness to purinergic agonists in specific subpopulations of patients. Thus, 19–26% of samples showed contractile responses to ATP and α,β- or β,y-methylene-ATP, which clearly exceeded the average. However, proving a concept of responders and non-responders depending on expression levels requires higher case numbers and is not possible on the basis of our data. Notably, even the highest purinergic contractions in presumed responders remained much weaker than neurogenic contractions, and purinergic contractions are probably no general component with relevant contributions to smooth muscle tone in the human prostate.

## Conclusions

Our findings suggest that ATP-induced smooth muscle contractions certainly occur in the human prostate, but point to a low relevance compared to other contractile stimuli in the human prostate and compared to purinergic contractions in non-human prostates. Purinergic contractions in the human prostate are not sensitive to NF023 or PPADS, suggesting that they may occur receptor-independently. To the best of our knowledge, our study represents the first study systematically addressing purinergic contractions in human prostate tissues.

## Supplementary information

ESM 1 (XLSX 11 kb)

ESM 2 (PZFX 22 kb)

ESM 3 (XLSX 439 kb)

ESM 4 (XLSX 29 kb)

ESM 5 (XLSX 75 kb)

ESM 6 (XLSX 53 kb)

ESM 7 (PZFX 23 kb)

ESM 8 (PZFX 25 kb)

ESM 9 (XLSX 102 kb)

## Data Availability

Original and raw data containing all individual data points are available as supplemental data, for experiments performed with organ bath and by RT-PCR. Complete blots are shown for western blot analyses in the manuscript, so that these blots are not included again in the supplementary data.
